# The Use of Intraoperative Extracorporeal Membrane Oxygenation in Lung Transplantation: Initial Institutional Experience

**DOI:** 10.21470/1678-9741-2021-0182

**Published:** 2023

**Authors:** Atakan Erkılınç, Mustafa Vayvada

**Affiliations:** 1 Anesthesiology and Reanimation Department, Kartal Kosuyolu Training and Research Hospital, Istanbul, Turkey.; 2 Thoracic Surgery Department, Kartal Kosuyolu Training and Research Hospital, Istanbul, Turkey.

**Keywords:** Lung Transplantation, Extracorporeal Membrane Oxygenation, Lung Disease, Survival

## Abstract

**Introduction:**

Lung transplantation is the final treatment option for end-stage lung disease, and extracorporeal membrane oxygenation (ECMO) is increasingly being used during lung transplantation.

**Objective:**

The present study aimed to review our initial experience with patients who underwent lung transplantation with or without ECMO since the implementation of the lung transplantation program at our center.

**Methods:**

Data were prospectively collected on all patients between December 2016 and December 2018. Patients undergoing ECMO as a bridge to lung transplantation were excluded.

**Results:**

A total of 48 lung transplants were performed, and ECMO was used in 29 (60.4%) cases. Twenty (83%) patients were female. The median age was 48.5 (range, 14-64) years. The most common indications were idiopathic interstitial pneumonia in 9 (31%) patients, chronic obstructive pulmonary disease in 7 (24.1%) patients, and bronchiectasis in 6 (20.7%) patients. Sequential bilateral lung transplantation was performed in all patients. The 30-day mortality was 20.6% (6/29) for patients with ECMO, however, it was 10.5 (2/19) for patients without ECMO (P=0.433). The median length of stay in the intensive care unit (ICU) was 5 (range, 2-25) days. The ECMO weaning rate was 82.8% (24/29). One-year survival was 62.1% with ECMO versus 78.9% without ECMO, and the 3-year survival was 54.1% versus 65.8%, respectively (P=0.317).

**Conclusions:**

ECMO is indicated for more severe patients who underwent lung transplantation. The use of ECMO provides adjuvant support during surgery and the mortality rate is acceptable. Survival is also as similar as non-ECMO patients. ECMO is appropriate for critically ill patients.

## INTRODUCTION

Lung transplantation is a well-established treatment option for end-stage lung diseases that do not respond to optimal medical therapy. The number of lung transplants has increased worldwide and the survival rates have also increased^[1]^. Lung transplantation can be performed with or without an extracorporeal life support system (ECLS). ECLS has been used for a long time to eliminate the deficiencies in the patient’s oxygenation supply or to overcome this process without facing any problems during lung transplant surgery^[2]^. Both strategies have advantages and disadvantages. Intraoperative ECLS can guarantee hemodynamic stability. The greatest benefit of ECLS is to avoid excessive volume overload and reperfusion damage to the first implanted lung. In addition, it can overcome oxygenation problems due to one-lung ventilation. The disadvantages of ECLS are risk of complications from arterial cannulation and bleeding due to anticoagulation therapy.

The use of cardiopulmonary bypass (CPB) during lung transplantation has dramatically decreased over time because of CPB-related bleeding due to coagulopathy, neurologic dysfunction, renal dysfunction, and induced systemic inflammatory response syndrome (SIRS) that may cause primary graft dysfunction (PGD)^[3]^. The use of extracorporeal membrane oxygenation (ECMO) in lung transplantation was first introduced in 2001 by Ko et al.^[4]^ and the first major series was presented by Aigner in 2007^[5]^. ECMO use in lung transplantation is increasing worldwide. Many centers prefer ECMO instead of CPB for intraoperative cardiac and pulmonary support^[3,5-7]^. Low heparinization, less bleeding requiring reoperation, possibility of continuous postoperative cardiopulmonary support, low SIRS rates, shorter intensive care unit (ICU) and hospital stays, lower PGD rates, and the opportunity for peripheral cannulation are some of the advantages of ECMO over CPB^[8]^.

In our clinic, ECMO is used for intraoperative cardiopulmonary support when indicated. In this study, we assessed the outcomes of intraoperative use of ECMO on mortality and survival of patients undergoing lung transplantation.

## METHODS

A total of 55 patients underwent lung transplantation between December 2016 and December 2018 at our center in Istanbul, Turkey. Our center is the leading transplantation center in this region. Seven patients who were on ECMO as a bridge to lung transplantation were excluded. CPB was not used for lung transplantation. Hence, 48 patients were included in the study cohort. Patients were divided into two groups based on the use of intraoperative ECMO. The patients’ demographic characteristics, preoperative data, intraoperative data, ICU length of stay, PGD, and survival rates were recorded. All data were prospectively recorded and retrospectively analyzed. PGD was defined according to the report of the International Society for Heart and Lung Transplantation Working Group on primary graft dysfunction in 2016. Severe primary graft dysfunction, defined as grade III, was considered when there was pulmonary edema on chest radiography and a PaO_2_ /FiO_2_ ratio <200 at 24, 48, and 72 hours postoperatively^[9]^. The patients’ clinical status was classified according to the Karnofsky Performance Scale (KPS). Patients with KPS scores <50 (inability to self-care; requiring institutional or hospital care or equivalent; rapid disease progression) were identified as those requiring complete assistance.

Pulmonary hypertension due to chronic lung disease (World Health Organization [WHO] Group 3) was defined as a mean pulmonary arterial pressure (mPAP) ≥25 mmHg measured by right heart catheterization with the patient in supine position and at rest. Pulmonary arterial hypertension (WHO Group 1) was defined as the precapillary pulmonary hypertension group with mPAP ≥25 mmHg, pulmonary capillary wedge pressure ≤15 mmHg, and pulmonary vascular resistance >3 Wood units^[10]^. Of our study population, 47 patients were in WHO Group 3.

### ECMO Indications and Cannulation Technique

**Fig. 1:**
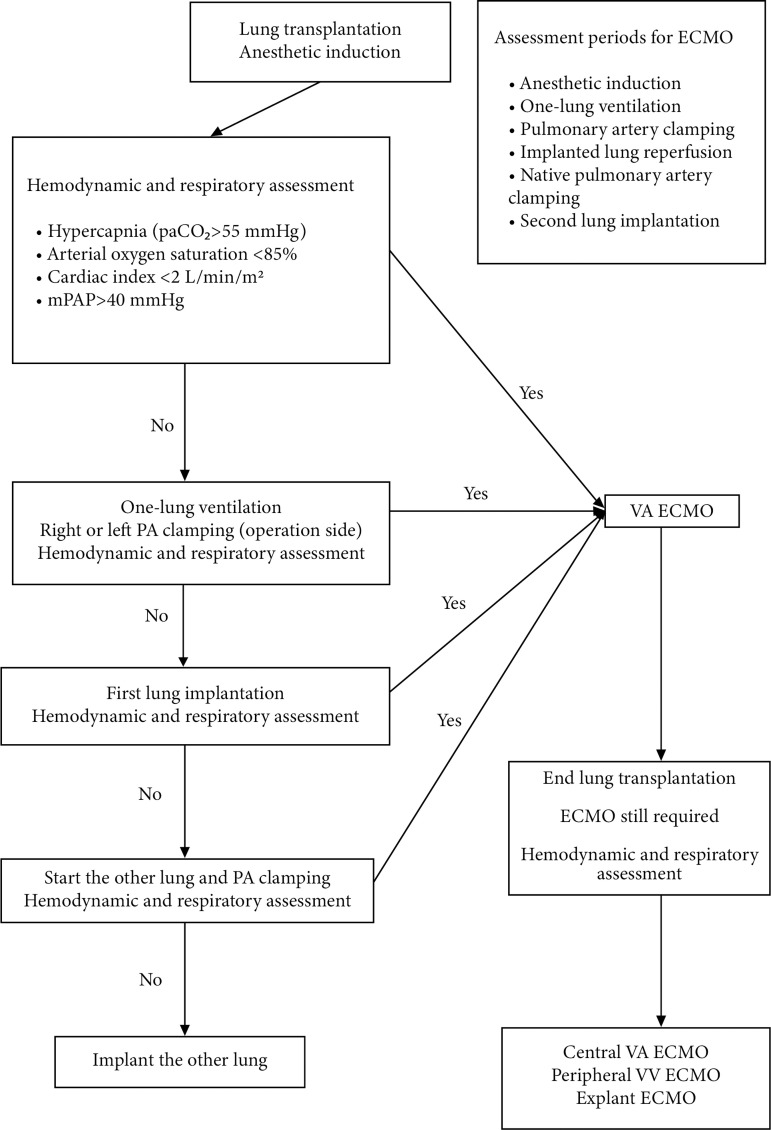
Algorithm for the use of intraoperative ECMO.

Our surgical procedure was sequential bilateral lung transplantation through clamshell incision. After incision, purse-string sutures were placed for cannulation from the right atrium and ascending aorta for establishing central venoarterial (VA) ECMO. The indication for the use of intraoperative ECMO was hypercapnia (paCO_2_ >55 mmHg). This criterion was based on transplant centers in Munich, Hanover, and Zurich and modified by our national lung transplant community^[11-13]^. After implantation of the first lung, the native pulmonary artery was clamped to assess whether the new lung could provide adequate oxygenation (Figure 1).

Central VA ECMO was used for intraoperative cardiopulmonary support by cannulating the right atrial appendage and the ascending aorta. A 15-19 French (Fr) arterial cannula was used for the aorta, and a 2-stage venous cannula or a 36 Fr curved-tip cannula was used for the right atrium. Cell savers were used for all patients. The target ECMO blood flow during transplantation was 50-70 mL/kg, and the fraction of inspired oxygen was 100%, adjusted as per hemodynamic parameters and gas exchange results. Heparin was initially administered intravenously at 50 IU/kg, and the activated clotting time (ACT) was targeted between 160 and 180 seconds. Our standard practice is to monitor ACT hourly, partial thromboplastin time (PTT) every 4-6 hours, prothrombin time (PT)/international normalized ratio (INR) and fibrinogen every 12 hours, and platelet count at least twice a day. Transfusion of platelets and packed red blood cells (pRBCs) was standard of care and products were given to maintain fibrinogen levels >150 mg/dL, platelets >80-100 1.000/mL, and hematocrit (HCT) of 25%. ECMO was gradually reduced and terminated after implantation of both lungs. ECMO support was discontinued in hemodynamically stable patients and with the following arterial blood measures: PaO_2_ >70 mmHg, PaCO_2_ 35-50 mmHg, tidal volume 6-10 mL/kg, positive end-expiratory pressure within the acceptable limits (10 cmH_2_O), and low pulmonary artery pressure without right ventricular failure. ECMO was prolonged when the ratio of mean pulmonary artery pressure to mean arterial pressure (mPAP/mAP) was <2/3. Central ECMO was switched to peripheral venovenous (VV) ECMO in case of graft dysfunction after transplantation. In case of hemodynamic instability, the patient was transferred to the ICU with central VA ECMO.

### Statistical Analysis

Distribution of quantitative data was analyzed using the Kolmogorov-Smirnov test. Normally distributed data are presented as mean±SD, not normally distributed as median (minimum-maximum). Qualitative data are presented as a percentage of the analyzed group. To compare characteristics between lung transplantation groups with and without ECMO, Student’s t-test and Mann-Whitney U-test were used, when appropriate. Qualitative data were compared using Fisher’s exact test and chi-square test. The Kaplan-Meier method was used for survival and the differences between lung transplantation with and without ECMO were analyzed with the log-rank test. A value of *P*<0.05 was considered statistically significant. The analysis was performed using the SPSS for Windows software (IBM, Armonk, New York, USA).

## RESULTS

A total of 48 patients were included in the study and divided into ECMO (Group A, n=29) and non-ECMO (Group B, n=19) groups. The primary diagnoses among patients were idiopathic interstitial pneumonia (20 [41.6%]), chronic obstructive pulmonary disease (9 [18.8%]), bronchiectasis (9 [18.8%]), cystic fibrosis (6 [12.5%]), silicosis (3 [6.3%]), and idiopathic pulmonary arterial hypertension (1 [2%]). Table 1 lists the indications for ECMO in each group. Idiopathic interstitial pneumonia was the most common diagnosis in both groups.

**Table 1:** Patient characteristics.

	Group A (n=29)	Group B (n=19)	*P*-value
Male sex	20 (83)	14 (82)	0.999
Age, years	48.5 (14-64)	53 (21-64)	0.474
BMI (kg/m^2^)	24.1 (13.7-32.2)	24.4 (18.6-29.4)	0.937
Diagnosis			
IIP	9 (31)	11 (57.9)	
COPD	7 (24.1)	2 (10.5)	
Bronchiectasis	6 (20.7)	3 (15.8)	
CF	4(13.8)	2 (10.5)	
Silicosis	2 (6.9)	1 (5.3)	
IPAH	1 (3.5)		
FVC (%)	34.5 (18-91)	35 (21-70)	0.427
FEV1 (%)	34.5 (13-92)	35 (13-73)	0.525
6-minute walk test, meters	85.5 (0-330)	90 (0-320)	0.841
DLCO (%)	32.5 (8-47)	38 (31-65)	0.022
KPS <50 (complete assistance)	19 (65.5)	11 (57.9)	0.594
Echocardiogram			
Dilated/hypertrophic RV	15 (62.5)	7 (46.6)	0.132
TAPSE, mm	19.5 (13-28)	18 (11-28)	
Right heart catheterization			
sPAP, mmHg	48 (12-78)	37 (25-49)	0.001
mPAP, mmHg	30.5 (21-95)	20 (15-35)	0.005
CO	5 (3.4-7)	4 (3.6-4.5)	0.233

Values are expressed as median (min.-max. range) or numbers and percentages. BMI=body mass index; IIP=idiopathic interstitial pneumonia; CF=cystic fibrosis; CO=cardiac output; COPD=chronic obstructive pulmonary disease; DLCO=diffusing capacity for carbon monoxide; FEV1=forced expiratory volume in the first second; FVC=forced vital capacity; KPS=Karnofsky Performance Status; mPAP=mean pulmonary artery pressure; RV=right ventricle; sPAP=systolic pulmonary artery pressure; TAPSE=tricuspid annular plane systolic excursion

Patient characteristics were similar between groups in terms of sex, age, body mass index (BMI), pulmonary function tests, and 6-minute walk test. The diffusion capacity of the lungs for carbon monoxide (DLCO) was significantly lower in Group A (*P*=0.022). Echocardiographic findings were not different between groups. Systolic and mean pulmonary artery pressures were higher in Group A than in Group B at the right heart catheterization (*P*=0.001 and *P*=0.005, respectively; Table 1).

Intraoperative data and outcomes are listed in Table 2. Cold ischemia time did not differ significantly between groups (*P*=0.054). In addition, donor variables were similar between the two groups. The need for red blood cell transfusion intraoperatively and on the 1^st^ postoperative day were more frequent in Group A (*P*=0.001). ICU stay, need for postoperative tracheostomy, and days on postoperative mechanical ventilation were higher in Group A (*P*=0.002, *P*=0.039, *P*=0.041, respectively; Table 2). ECMO-related complications were: mediastinal cannulation site bleeding in 1 patient (3.4%), surgical site bleeding in 3 patients (10.3%), renal replacement therapy required in 3 patients (10.3%), cardiac arrhythmia in 6 patients (20.6%).

**Table 2:** Intraoperative data and outcomes.

	Group A (n=29)	Group B (n=19)	*P*-value
Transplantation type			
Single		2 (10.5)	
Bilateral/lobar	24 (82.8)/5 (17.2)	17 (89.5)	
Waiting time (days)	109 (4-433)	95 (5-206)	0.548
Donor variables			
Cause of death			
Intracranial bleed/stroke	12 (41.4)	11 (57.9)	
Trauma	12 (41.4)	6 (31.6)	
Unknown cardiac arrest	2 (6.9)	1 (5.2)	
Gunshot wound	2 (6.9)	1 (5.2)	
Alcohol intoxication	1 (3.4)		
Age	36 (16-55)	40 (19-54)	0.650
PaO_2_ mmHg on FiO_2_ of 1.0	380 (232-592)	341 (220-660)	0.195
Intubation time (days)	3 (1-6)	4 (1-9)	0.860
Heavy smoker (>20 pack-years)	5 (17.2)	6 (20.7)	0.304
Ischemic time (min)			
First lung	355 (310-480)	310 (270-450)	0.054
Second lung	505 (420-620)	480 (360-425)	
Number of transfusions on 1^st^ postoperative day, units			
RBC			
FFP	8 (3-21)	5 (0-7)	0.001
Platelets	6 (3-15)	5 (0-15)	0.286
	1 (0-7)	1 (0-6)	0.499
Postoperative ECMO	5 (17.2)	1 (5.2)	0.113
Severe PGD	11 (37.9)	6 (31.6)	0.653
Mechanical ventilation, days	3 (1-19)	1 (1-6)	0.041
Tracheostomy	8 (33.3)	1 (5.8)	0.039
Complications			
Mediastinal cannulation site bleeding	1 (3.4)		
Surgical site bleeding	3 (10.3)		
Renal replacement therapy required	3 (10.3)		
Cardiac arrhythmia	6 (20.6)		
ICU stay, days	5 (2-25)	3 (1-11)	0.002
Hospital discharge	20 (68.9)	17 (89.5)	0.152
30-day mortality	6 (20.6)	2 (10.5)	0.433
Cause of death			
Bleeding	2	-	
Cardiac failure	2	-	
Sepsis	1	1	
Myocardial infarction	1	-	
Hyperacute rejection	-	1	
Survival			
1 year (%)	62.1	78.9	
3 years (%)	54.1	65.8	0.317

Values are expressed as median (min.-max. range) or numbers and percentages. ECMO=extracorporeal membrane oxygenation; FFP=fresh frozen plasma; ICU=intensive care unit; PGD=primary graft dysfunction; RBC=red blood cells

ECMO support was extended into the postoperative period in 5 patients (17.2%) in Group A. The mean duration of postoperative ECMO support was 7 days (3-19 days). Four patients died and 1 patient survived. One patient was switched to peripheral VV ECMO from central VA ECMO because of graft dysfunction, and ECMO support was weaned off after 5 days. The patient was alive at the 18^th^ months of follow-up. Central VA ECMO was extended in 4 patients owing to hemodynamic instability; however, ECMO weaning was unsuccessful in all these patients. Two patients died from bleeding on the 3^rd^ postoperative day, 1 patient died because of cardiac failure on the 5^th^ postoperative day, and the fourth patient received a diagnosis of idiopathic pulmonary arterial hypertension and died on the 19^th^ postoperative day from right heart failure. In Group B, hyperacute rejection was observed postoperatively in 1 patient, and peripheral VV ECMO was performed on postoperative day 0; however, the patient died on the 7^th^ postoperative day.

In Figure 1, we have indicated the periods in which we evaluated the use of intraoperative ECMO. Apart from these periods, unplanned ECMO was applied to six patients. Although there was no requirement for ECMO in the evaluation period before the second lung anastomosis, 3 patients developed hypoxia that could not be corrected with ventilator strategies after the anastomosis started. In 1 patient, during first-side pneumonectomy, severe hypercarbia occurred that induced acidosis and myocardial depression. Severe hemodynamic instability developed in 2 patients due to possible right ventricular failure that could not be corrected with inotropic support during left-side anastomosis.

**Fig. 2:**
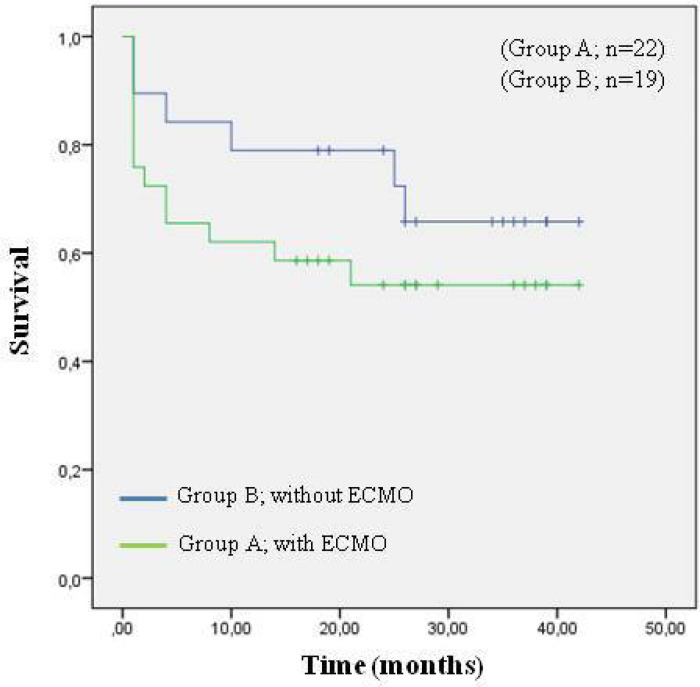
Overall survival.

The overall mortality was 16.6% (n=8) in our study. No significant difference was observed in 30-day mortality between the two groups (20.6% *vs.* 10.5%, *P*=0.433). The rate of hospital discharge was lower in Group A than in Group B (68.9% *vs.* 89.5%, *P*=0.152). All patients from Group B were discharged; however, 3 patients from Group A who were not discharged died 3, 4, and 6 months after lung transplantation. The 1- and 3-year survival rates did not differ significantly between groups: the 1-year survival rate was 62.1% in Group A *versus* 78.9% in Group B, and the 3-year survival rate was 54.1% in Group A *versus* 65.8% in Group B (*P*=0.317, Figure 2).

## DISCUSSION

The preliminary outcomes of our study revealed that patients with low DLCO values and pulmonary hypertension required ECMO during lung transplantation. In addition, prolonged ICU stay and long-term rehabilitation were required for these patients. Tracheostomy was performed more frequently because of prolonged ventilation time. However, mortality and overall survival did not differ between ECMO and non-ECMO groups.

Lung transplantation is indicated for end-stage lung disease and patients with low lung compliance may require ECMO during surgery. Clamping the pulmonary artery for 3-5 min before pneumonectomy is a common practice. If hemodynamic instability occurs or the pulmonary artery pressure increases, ECMO should be established immediately. In addition, the heart is retracted caudally during left lung anastomosis, and this maneuver can lead to right heart failure and deterioration. ECMO is indicated during this step to overcome the malposition of the heart.

Intraoperative circulatory support is required for cardiopulmonary instability during transplantation. ECMO is used more frequently over CPB given the lower risk of bleeding complications and PGD as well as improved overall survival^[7,14]^. The frequency of ECMO use varies across different centers; however, ECMO is primarily used in patients with high oxygen deficiency and severe hemodynamic instability. At our center, we assess patients for ECMO use during anesthetic induction and during one-lung ventilation, pulmonary artery clamping, reperfusion phase, and second lung implantation.

We prefer VA ECMO at our clinic because cardiac output with VV ECMO has been reported to be inadequate in patients with right ventricular dysfunction. VA ECMO, in addition to providing hemodynamic stability, reduces pulmonary perfusion pressure by helping bypass a significant portion of cardiac output to the pulmonary vascular bed, thereby preventing tissue shear stress and endothelial damage in the pulmonary vessels. With central VA ECMO, higher blood flow can be maintained. In addition, oxygenated blood access to coronary and cerebral vessels will reduce the overall mortality. Patient mortality did not depend on ECMO configuration, but general ECMO imperatives like anticoagulation and cannulation complications may affect morbidity by increasing bleeding and requiring more blood products. In patients with pulmonary hypertension or right heart failure, assessment for ECMO use should be performed in the preoperative period to prevent complications that may require emergency interventions. The use of ECMO as an important support device in patients with idiopathic pulmonary arterial hypertension has been well established^[15]^. Patients who underwent unplanned intraoperative ECMO had poorer prognosis than those who underwent planned intraoperative ECMO^[16]^. Unplanned intraoperative ECMO was performed in 6 patients, 4 of whom died.

The effects of ECMO on PGD remain unclear. Pulmonary artery clamping of the native lung during the second lung transplant leads to shear stress in the vascular bed of the implanted lung due to the passage of the entire cardiac output, thereby causing primary graft dysfunction^[16]^. Ius et al.^[13]^ reported that the incidence of PGD was higher among patients receiving ECMO. In contrast, VA ECMO has been shown to prevent the development of PGD by diverting blood circulation to pulmonary vascular structures and affecting cell rheology with the heparin administered during ECMO^[13]^. Per our results, the incidence of severe PGD was similar in both groups. ECMO use was not an additive risk factor for patients at high risk of PGD (higher pulmonary artery pressure and use of more blood products).

Perioperative management and postoperative care are pertinent concerns for ECMO support strategy for lobar lung transplantation, which differs from that for standard lung transplantation. After implantation of the lobe and during the other native lung pneumonectomy, nearly the entire cardiac output is routed to the lobe. This excessive pulmonary circulation leads to higher pulmonary vascular pressure, causing extravascular fluid leakage and eventually pulmonary edema. ECMO support is recommended during surgery to prevent overloading of the pulmonary vascular bed^[17]^. Patients who are candidates for lobar lung transplantation are at a higher risk of hemodynamic instability during the perioperative reperfusion stage because of the poor overall condition of the recipients. Lobar lung transplantation was performed in 5 patients, and ECMO was used in all of them. Mortality occurred in 1 patient because of massive bleeding.

In cases where ECMO support is necessary, bleeding after heparinization is the most challenging surgical complication, particularly in patients with pleural thickening, pleurodesis, and previous thoracotomy. Hoetzenecker et al.^[18]^ reported that the use of erythrocyte suspension (ES) was greater in patients who received ECMO than in patients who did not receive ECMO. Consistent with this finding, in our study, the use of ES was significantly higher in the ECMO group. The center’s experience, however, is important in this regard. Our preliminary results revealed that a higher proportion of patients with pleural adhesions underwent lung transplantation with a higher need for blood transfusion.

In our study, the 1- and 3-year survival rates were lower in patients in the ECMO group, although the difference was not significant. The Vienna transplant team^[18]^ reported higher survival rates in the ECMO group, while the Zurich transplant team^[12]^ reported higher survival rates in the non-ECMO group. The 1-year survival rates in the ECMO *versus* non-ECMO groups were 84.2% *versus* 90.4% for the Zurich transplant team and 91% *versus* 82% for the Vienna transplant team, respectively, and the 5-year survival rates were 52.8% *versus* 70.5% and 80% *versus* 74%, respectively.

The present study has several limitations. This was a retrospective study and, owing to limited experience with lung transplantation in our region, our sample size was small. In addition, the mean transplant waiting time for all patients was only 103 days. During the study period, we performed 55 lung transplants, and 34 patients died on the waiting list. Most of our study patients were on the borderline for the requirement of ECMO support. Therefore, we could not determine which patients would need ECMO as per their preoperative findings, except for increased pulmonary artery pressure during right heart catheterization and DLCO. Of all patients who underwent lung transplantation, 62.5% (30/48) were classified as requiring complete assistance as per the KPS score. This explains the use of ECMO in most of our patients and a relatively high mortality compared to experienced centers.

## CONCLUSION

In conclusion, our experience shows that ECMO should be used frequently during a new lung transplant program in a center with a limited number of donors and patients with poor overall status. Pulmonary hypertension and low DLCO are indicators for intraoperative ECMO support. Patients who undergo unplanned intraoperative ECMO have a worse prognosis. Greater use of blood products was required in patients receiving ECMO support. Although patients who required intraoperative ECMO support had worse outcomes, the use of intraoperative ECMO helps to perform lung transplantation in high-risk patients.
